# An AP2/ERF Gene, *HuERF1*, from Pitaya (*Hylocereus undatus*) Positively Regulates Salt Tolerance

**DOI:** 10.3390/ijms21134586

**Published:** 2020-06-28

**Authors:** Yujie Qu, Quandong Nong, Shuguang Jian, Hongfang Lu, Mingyong Zhang, Kuaifei Xia

**Affiliations:** 1 Key Laboratory of South China Agricultural Plant Molecular Analysis and Genetic Improvement, Guangdong Provincial Key Laboratory of Applied Botany, South China Botanical Garden, Chinese Academy of Sciences, Guangzhou 510650, China; abcquyujie@163.com (Y.Q.); nongquand@163.com (Q.N.); 2 College of Life Sciences, University of Chinese Academy of Sciences, Beijing 100049, China; 3 Wenshan Academy of Agricultural Sciences, Wenshan 663000, China; 4 Engineering Laboratory for Vegetation Ecosystem Restoration on Islands and Coastal Zones, South China Botanical Garden, Chinese Academy of Sciences, Guangzhou 510650, China; jiansg@scbg.ac.cn (S.J.); luhf@scbg.ac.cn (H.L.); 5 Innovation Academy of South China Sea Ecology and Environmental Engineering, Chinese Academy of Sciences, Guangzhou 510650, China; 6 Center of Economic Botany, Core Botanical Gardens, Chinese Academy of Sciences, Guangzhou 510650, China

**Keywords:** *HuERF1*, ethylene, salt stress, pitaya, *Arabidopsis*

## Abstract

Pitaya (*Hylocereus undatus*) is a high salt-tolerant fruit, and ethylene response factors (ERFs) play important roles in transcription-regulating abiotic tolerance. To clarify the function of *HuERF1* in the salt tolerance of pitaya, *HuERF1* was heterogeneously expressed in *Arabidopsis*. *HuERF1* had nuclear localization when *HuERF1::GFP* was expressed in *Arabidopsis* protoplasts and had transactivation activity when *HuERF1* was expressed in yeast. The expression of *HuERF1* in pitaya seedlings was significantly induced after exposure to ethylene and high salinity. Overexpression of *HuERF1* in *Arabidopsis* conferred enhanced tolerance to salt stress, reduced the accumulation of superoxide (O_2_$\bar{\cdot }$) and hydrogen peroxide (H_2_O_2_), and improved antioxidant enzyme activities. These results indicate that *HuERF1* is involved in ethylene-mediated salt stress tolerance, which may contribute to the salt tolerance of pitaya.

## 1. Introduction

Pitaya belongs to the Cactaceae family and genus *Hylocereus*. Pitaya fruits have high nutritional and economical values and they are mainly cultivated in tropical and subtropical zones. Pitaya has a high tolerance to abiotic stress [1], but adverse environments such as salt, drought, and high temperature still limit pitaya’s growth and yield productivity. Approximately 20% of the irrigated farmland worldwide is affected by salt stress in the world [2]. Recently, the molecular mechanisms associated with pitaya response to salt and drought stresses were explored at the transcriptomic and proteomic levels [2,3]. It is rather important that the crop is developed to improve tolerance to salt stress. To date, only a few stress-associated genes, including *miR396b-GRF* and *HuCAT3*, in pitaya have been reported to enhance tolerance to cold, drought, and salt stress [4,5].

Ethylene (C_2_H_4_) plays an important role in plant growth, development, and stress responses [6]. Ethylene signaling-mediated salt stress response and the mechanism of salt stress response have been primarily disclosed [7,8]. *Arabidopsis EIN2* and *EIN3* mutants exhibit the enhanced salt stress sensitivity, indicating that *EIN2* and *EIN3* are positive regulators in the ethylene-mediated salt stress response [8,9].

As a crucial regulator of ethylene signaling, APETALA2/ethylene responsive factor (AP2/ERF) is involved in plant growth and development, primary and secondary metabolism, as well as responses to environmental stresses [10]. AP2/ERF proteins include at least one AP2 domain, the DNA binding domain, and they have been divided into three separate families, namely, AP2, ERF, and RAV. The ERF family contains a single AP2 domain, and the AP2 family contains one or two AP2 domains [11]. The AP2/ERF domain consists of about 60–70 amino acid residues that confer a typical three-stranded anti-parallel β-sheet and an α-helix [12]. In the ERF family, the AP2/ERF DNA binding domain has been divided into DREB and ERF domains according to the characteristics of residues at specific positions [13]. Many DREB proteins have been proved to be bound with an A/GCCGAC motif, which is often related to drought, ABA, and cold-responsive genes [14]. In contrast, proteins of the ERF subfamily specifically bind an AGCCGCC motif, namely, the GCC-box, which responds to ethylene, wounding, and pathogens [15]. In addition, DREB subfamily proteins contain conserved amino acid residues Val and Glu at positions 14 and 19, respectively, of the AP2/ERF domain, and the ERF subfamily protein contains Ala and Asp at the corresponding positions [16]. There are many reports that stress-responsive AP2//ERF genes enhance the tolerance of plants to stress. *IbRAP2-12* was cloned from sweet potato, and ectopic expression of *IbRAP2-12* improved tolerance to salt and drought stresses in transgenic *Arabidopsis* [17]. Overexpression of soybean *GmERF3* increased tolerance to salt and drought stresses in transgenic tobacco [18]. Overexpression of *AtERF1* in *Arabidopsis* significantly enhanced tolerance to salt and drought stresses [19]. Peanut *AhERF019*-overexpressing *Arabidopsis* lines increased tolerance to different stresses [20]. *Arabidopsis AtERF15* positively regulates ABA and stress responses [21]. *ZmERF1* was isolated from maize (*Zea mays* L.), and its expression could be highly induced by salt stress, indicating that the ZmERF1 protein plays vital roles in abiotic stress tolerance [22]. Some salt stress-responsive genes have been identified in pitaya by transcriptome analysis [23].

The *HuERF1* was continuously highly induced after salt treatment in our previous transcriptomic result. To clarify the function of *HuERF1* in the salt tolerance of pitaya, *HuERF1* was isolated from pitaya and overexpressed in *Arabidopsis*. Overexpression of *HuERF1* in *Arabidopsis* could enhance salt tolerance in *Arabidopsis*, and it may contribute to the high salt tolerance of pitaya and help us understand why pitaya is salt tolerant.

## 2. Results

### 2.1. Sequence and Phylogenetic Analyses of HuERF1

**Figure 1:**
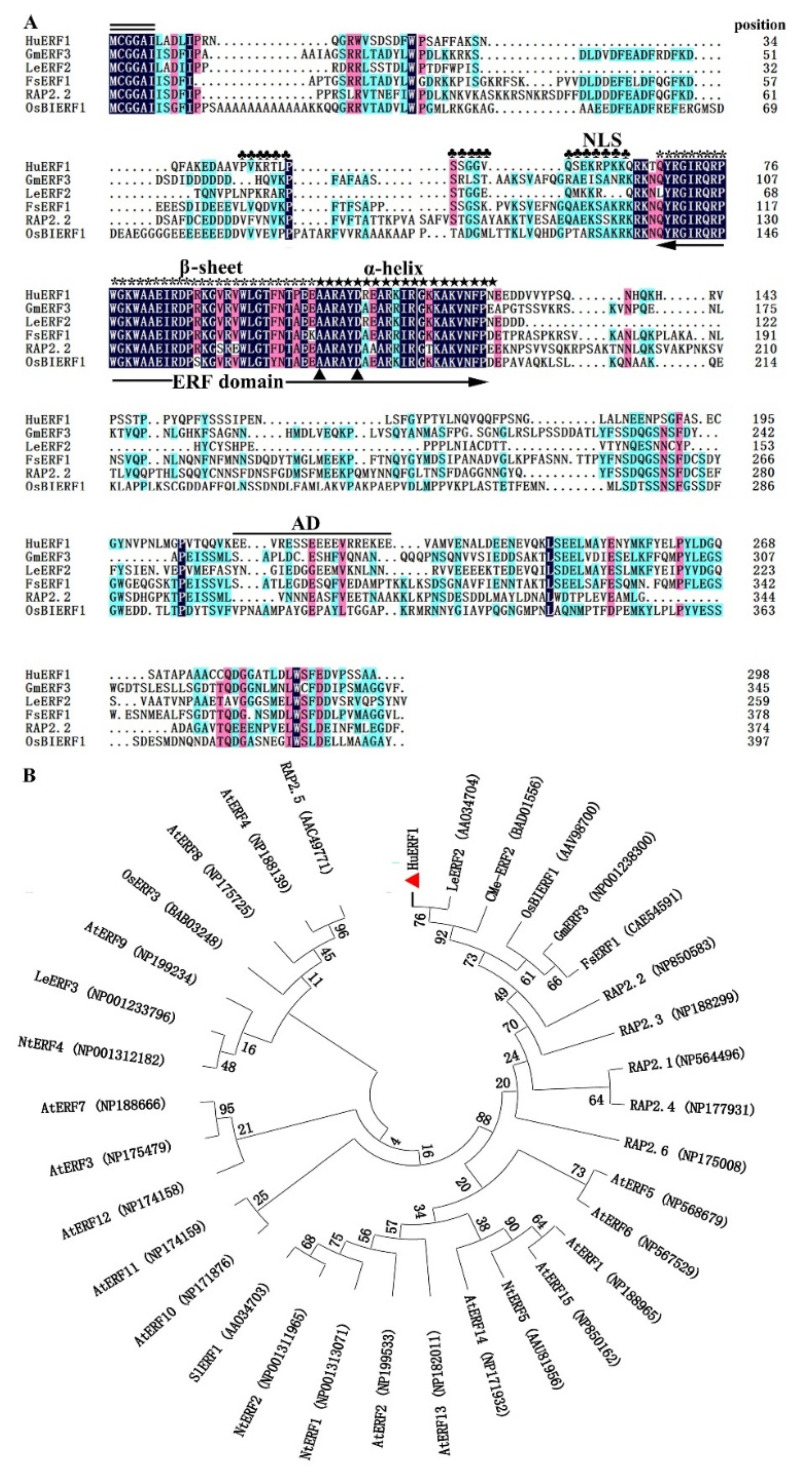
Sequence analysis of HuERF1 and phylogenetic relationship of HuERF1 with other ethylene response factor (ERF) proteins. (**A**) Comparison of the deduced amino acid sequences of AP2/ERF-related proteins that have high sequence similarity with HuERF1. Identities and similarities among amino acid sequences are colored. Double black bars above the sequence represent the highly conserved N-terminal MCGGAII/L motif of unknown function. The single black bar above the sequence represents the putative acidic domain (AD). Plum flowers (♣) represent putative nuclear localization signals. The asterisks (*) represent β sheets of a conserved DNA-binding domain (AP2/ERF domain). Five-pointed stars (★) represent α helix. Dots (.) show gaps in the amino acid sequences introduced to optimize alignment. Two triangles (▲) indicate the 14th Ala and 19th Asp positions of the AP2/ERF domain. The AP2/ERF domain is indicated by double arrows. The amino acid sequences are shown as follows: GmERF3 (NP001238300), LeERF2 (AAO34704), FsERF1 (CAE54591), RAP2.2 (NP850583), and OsBIERF1 (AAV98700). (**B**) A phylogenetic tree of ERF subfamily proteins based on MEGA 7.0 software. The results show the relative similarity of the full-length HuERF1 protein in pitaya and AP2/ERF proteins from other species.

Among 29 ERF homologs identified in piatya transcriptome, *HuERF1* was continuously highly induced by salt treatment. Therefore, the full-length open reading frame (ORF) of *HuERF1* was obtained from salt-treated pitaya seedlings by RT-PCR. It is 897 bp and predicted to encode a protein of 298 amino acid residues. The predicted protein consists of an AP2/ERF domain with 59 amino acids (68–126) in the N-terminal region and an acid domain consisting of 18 amino acid (212–229) (Figure 1A). In addition, a putative nuclear localization signal (NLS) (44–64) was detected in the N-terminal of the protein, which is a feature of the ERF subfamily [12]. Furthermore, *HuERF1* belongs to the ERF subfamily and shows the closest relationship to LeERF2 from tomato based on phylogenetic analysis (Figure 1B).

### 2.2. Ethylene Improved Pitaya Plant Tolerance to Salt Stress

**Figure 2:**
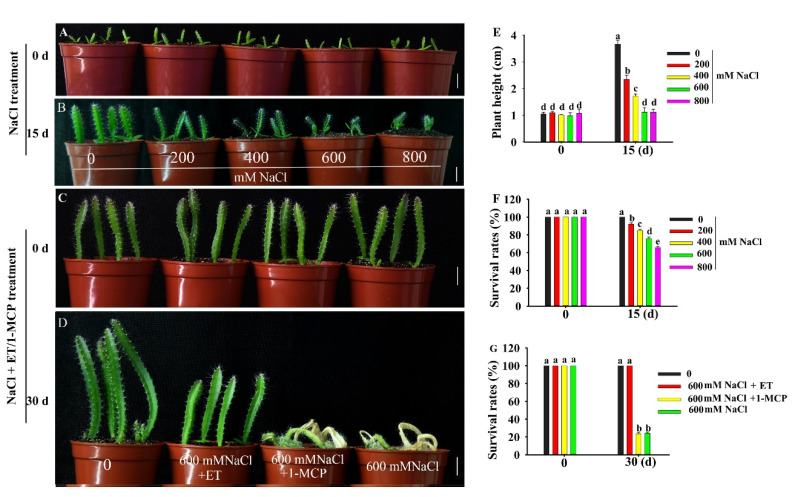
Phenotype of pitaya seedlings under salt, ET (ethephon) and 1-MCP (1-methylcyclopropene) treatments. (**A**,**B**,**E**,**F**) Phenotype (**A**,**B**), plant height (**E**) and survival rate (**F**) of two-week-old pitaya seedlings treated with different concentrations of NaCl for 15 days. (**C**,**D**,**G**) Phenotype (**C**,**D**) and survival rate (**G**) of six-week-old pitaya seedlings treated with 600 mM NaCl as well as 100 µM ET or 100 µM 1-MCP for 30 days. Data are presented as the mean ± SE (*n* = 3). Different letters (**E**–**G**) indicate a significant difference from that of the control at *p* < 0.05, ANOVA followed by Fisher’s LSD test. Bars = 1.5 cm.

Pitaya could tolerate relatively higher salt stress conditions. When two-week-old pitaya seedlings were treated with different concentrations of NaCl (Figure 2B), some of them survived after treatment for 15 days under 800 mM NaCl (Figure 2B,F). However, there was an obvious phenotypic difference between control plants and plants treated with different concentrations of NaCl (Figure 2B). With the increase in salt concentrations, plant height and survival rates decreased (Figure 2E,F). Compared with the control, the plant heights of pitaya seedlings treated with 200, 400, 600, and 800 mM NaCl were reduced by 1.22, 1.71, 2.51, and 2.71 cm, respectively, and the survival rates were reduced by 7.81%, 15.06%, 24.13%, and 34.25%, respectively.

Ethephon (ET, ethylene compound) could improve the tolerance of pitaya seedlings to salt stress (Figure 2D). When six-week-old pitaya seedlings were treated with 600 mM NaCl as well as 100 µM ET or NaCl alone for 30 days, the survival rate of pitaya seedlings under the condition of 600 mM NaCl as well as 100 µM ET remained at 100% and was higher than that, 24.19%, under 600 mM NaCl alone (Figure 2D,G). In contrast, when six-week-old pitaya plants were treated with 600 mM NaCl as well as 100 µM 1-MCP (1-methylcyclopropene, ethylene inhibitor) for 30 days, the survival rate of the seedlings under NaCl and 1-MCP treatments was 23.5% (Figure 2D,G). These results indicated that pitaya has high salt tolerance and ET can enhance its salt tolerance.

### 2.3. Expression of HuERF1 was Induced by Salt and Ethylene

To explore the expression pattern of *HuERF1*, different tissues were sampled from pitaya plants at the flowering stage, and qRT-PCR was performed (Figure S1). The result indicated that *HuERF1* was differentially expressed in various tissues. The expression levels of *HuERF1* were the highest in roots and the lowest in both squamas and stems.

**Figure 3:**
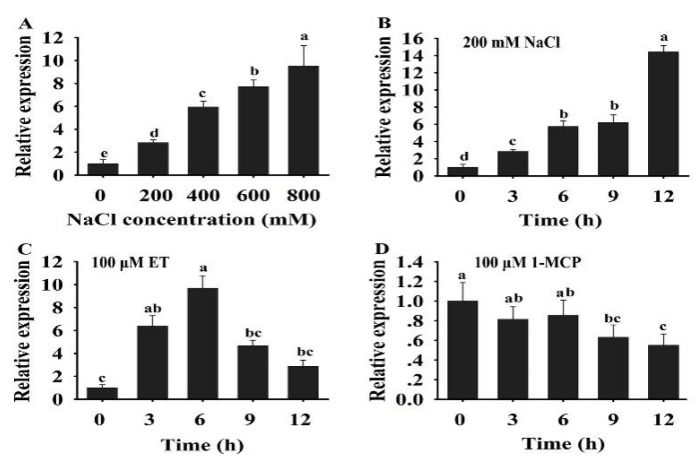
Expression analysis of *HuERF1* under salt and ethylene treatments. (**A**) Expression pattern of *HuERF1* in response to different concentrations of NaCl. Two-week-old pitaya seedlings were treated with different concentrations of NaCl (0, 200, 400, 600, and 800 mM). Total RNA was extracted from pitaya seedlings after NaCl treatment for 3 h and used for qRT-PCR. (**B–D**) Expression analysis of *HuERF1* in response to NaCl, ET, and 1-MCP treatments. Roots of two-week pitaya seedlings were dipped in a mock solution or 200 mM NaCl (**B**), 100 μM ET (**C**), and 100 μM 1-MCP (**D**), and the seedlings were sampled after 0–12 h of exposure for qRT-PCR. The value at 0 h was set to 1. *HuEF1-α* was used as the internal standard. Different letters indicate a significant difference at *p* < 0.05, ANOVA followed by Fisher’s LSD test. Mean values and SDs of three biological replicates are shown.

In plants, ERFs play a key role in response to multiple signal stimulation. To investigate the potential role of *HuERF1* in the response to salt stress and ethylene, the expression profiles of *HuERF1* were analyzed under salt stress, ethylene, and 1-MCP. The expression of *HuERF1* was dramatically induced after salt and ET treatments (Figure 3A–C), but its expression was suppressed by 1-MCP treatments (Figure 3D). Among different concentrations of salt, the expression level of *HuERF1* was the highest at 800 mM NaCl (Figure 3A). With respect to the time courses after 200 mM NaCl treatment, the expression of *HuERF1* gradually increased and reached its peak at 12 h (Figure 3B). In the 100 μM ET treatment, the expression level of *HuERF1* rapidly increased and reached a peak at 6 h, and then decreased to a normal level (Figure 3C). In the 100 μM 1-MCP treatment, the expression of *HuERF1* was downregulated in pitaya plants during a 12 h period and reached the lowest point at 9 h (Figure 3D). These results indicated that *HuERF1* expression was induced by salt and ethylene.

### 2.4. Overexpression of HuERF1 in Arabidopsis Improved Tolerance to Salt stress

To prove the role of *HuERF1* in salt tolerance, transgenic *Arabidopsis* overexpressing *HuERF1* under the control of the *CaMV35S* promoter was generated. Sixty eight transgenic positive transgenic plants were obtained at T_1_ lines by PCR and kanamycin identification and most of the transgenic lines accord with the segregation ratio (sensitive: resistant = 1:3). Two independent homozygous *HuERF1* lines (OE3 and OE4) with high expression levels at T2 and T3 lines were selected for further study (Figure S2).

**Figure 4:**
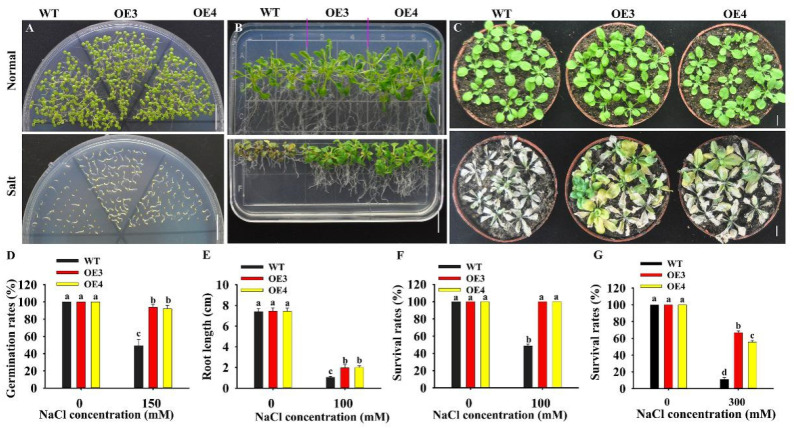
Overexpression of *HuERF1* enhances *Arabidopsis* tolerance to salt stress. (**A,D**) Overexpression of *HuERF1* improves the seed germination of *Arabidopsis*. Seed germination of *HuERF1-*overexpressing *Arabidopsis* (OE) on MS medium with 0 or 150 mM NaCl for 6 days (**A**), and germination rates (**D**) were measured. (**B**,**E**,**F**) Overexpression of *HuERF1* improves the tolerance of *Arabidopsis* growth to salt stress on MS medium. *Arabidopsis* seedlings were grown on MS medium with 0 or 100 mM NaCl for 15 days, root length (**E**) and survival rates (**F**) were measured. (**C**,**G**) Overexpression of *HuERF1* improves the tolerance of *Arabidopsis* growth to salt stress in pots. The seedlings were grown in pots for 3 weeks with 300 mM NaCl, survival rates (**G**) were measured. Different letters (**D**–**G**) indicate a significant difference at *p* < 0.05, ANOVA followed by Fisher’s LSD test. Mean values and SDs of three biological replicates are shown. Bars = 1 cm.

To test the role of *HuERF1* in seed germination under salt stress, the seeds of the *HuERF1-*overexpressing *Arabidopsis* were germinated on Murashige and Skoog’s medium (MS medium) containing 0 and 150 mM NaCl. The seed germination rates of OE3 and OE4 were 43.69 % higher than that of WT under 150 mM NaCl, while there were no significant differences between them under normal condition (Figure 4A,D). The *HuERF1-*overexpressing *Arabidopsis* grew better than WT plants on MS medium supplemented with 100 mM NaCl (Figure 4B). In pot growth under salt stress, OE3 and OE4 also showed significantly higher tolerance to 300 mM NaCl for 3 weeks (Figure 4C,G). These results indicated that *HuERF1* could improve salt tolerance in transgenic *Arabidopsis* in terms of both seed germination and growth. In addition, the OE lines seem to grow a little faster than the wild type in MS after 15 days planting (Figure 4B).

### 2.5. HuERF1 Exhibited Transcriptional Activity and Nuclear Localization 

**Figure 5:**
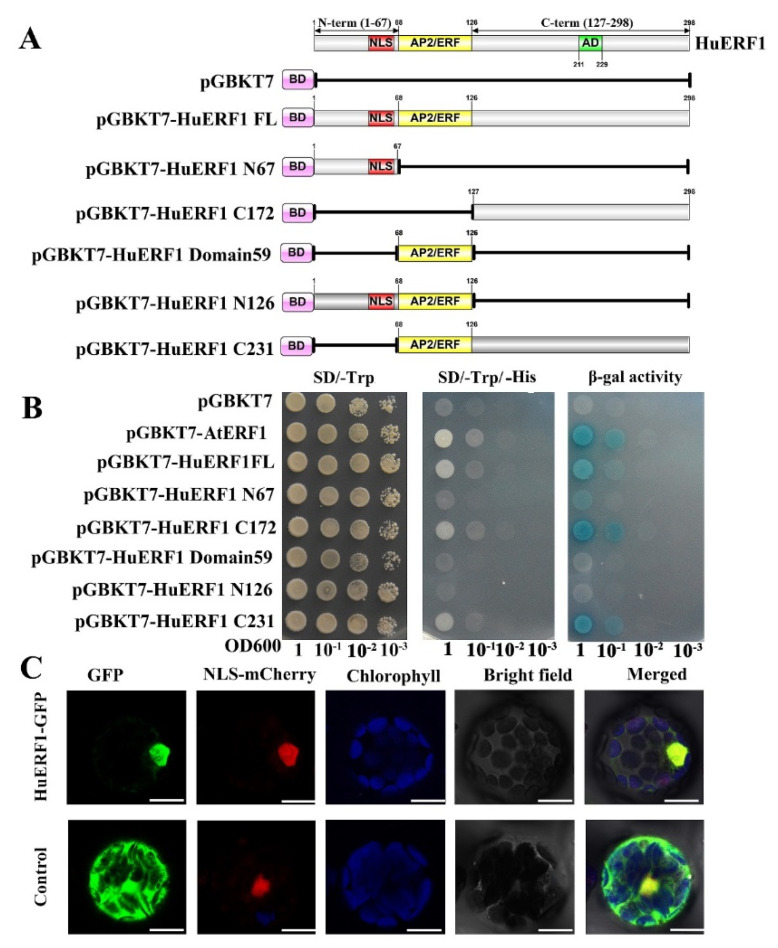
Transcriptional activation activity and nuclear localization of HuERF1. (**A**) Transactivation activity analysis of the GAL4 DNA binding domain-HuERF1 fusion assay in yeast. The GAL4 DNA binding domain was fused with the full-length and truncated forms of HuERF1 and transformed into the yeast strain AH109. (**B**) Analysis of activity of the yeast strains on plates. The yeast AH109 containing plasmids that encode GAL4-HuERF1 was cultured on SD/-Trp (left), SD/-Trp/-His (middle), and α-galactosidase activity (right). The yeast culture was serially diluted to OD_600_ values of 0.1, 0.01, 0.001, and then the 5-µL yeast liquid was spotted on SD plates and incubated for 4 days at 30 °C. (**C**) Nuclear localization of HuERF1. Subcellular localization of HuERF1 in *Arabidopsis* protoplasts observed under a laser scanning confocal microscope. The blue color indicates the autofluorescence emitted by chloroplasts; the red color indicates the nucleus using NLS-mCherry as the nuclear marker. Bars = 10 µm.

To investigate the transcription regulator activity of HuERF1 in yeast strain AH109, we exploited the deletion mutation of *HuERF1* to detect whether HuERF1 acts as a transactivation factor (Figure 5A,B). Yeast cells harboring the C172-terminal region, C231-terminal region, pGBKT7-AtERF1, and pGBKT7-HuERF1 grew well in the selection medium and exhibited β-galactosidase activity, suggesting the activation of the reporter gene, but the cells containing the N68-terminal region, N126-terminal region, empty vector pGBKT7 and domain 59 region failed to grow (Figure 5A,B). Therefore, these findings demonstrated that HuERF1 exhibited transcriptional activation activity and the C172-terminal region of HuERF1 acted as a potential transcriptional activator.

To explore the subcellular location of HuERF1 in vivo, *HuERF1-GFP* was used to perform a transient expression assay in *Arabidopsis* protoplasts (Figure 5C). The GFP fluorescence of HuERF1-GFP was mainly located in the nucleus, whereas the GFP protein was evenly distributed in the whole protoplast cells. The result indicated that HuERF1 is a nuclear-localized protein. 

### 2.6. Overexpression of HuERF1 Improved ROS Scavenging

**Figure 6:**
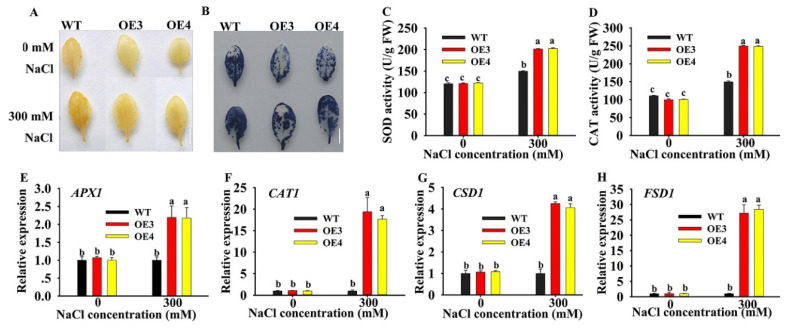
Oxidative stress analysis of *HuERF1*-overexpressing *Arabidopsis*. (**A**,**B**) Staining assays were carried out to detect H_2_O_2_ and O_2_$\bar{\cdot }$ by DAB (**A**) or NBT (**B**) staining, respectively. (**C**,**D**) The activity of CAT and SOD under salt treatment for 24 h. (**E–H**) Expression levels of the reactive oxygen species-related genes under salt stress for 24 h based on qRT-PCR. *AtAct2* was used as the internal standard. Different letters (**C**–**F**) indicate a significant difference at *p* < 0.05, ANOVA followed by Fisher’s LSD test. Mean values and SDs of three biological replicates are shown. Bars = 1 cm.

Most abiotic stresses lead to overaccumulation of reactive oxygen species (ROS) in plants, with damage caused to DNA, proteins, carbohydrates, and lipids, ultimately resulting in oxidative stress [24]. To explore the potential roles of *HuERF1* in oxidative stress. DAB (3, 3’-diaminnobenzidine) and NBT (nitro-blue tetrazolium) stains were used to detect H_2_O_2_ and O_2_$\bar{\cdot }$ accumulation levels in leaves of *HuERF1*-overexpressing *Arabidopsis* (Figure 6A,B). The staining assay indicated that OE3 and OE4 accumulated less H_2_O_2_ and O_2_$\bar{\cdot }$ than the WT. CAT and SOD Activities in OE3 and OE4 were significantly higher than those in the WT under salt stress conditions (Figure 6C,D). These findings indicated that overexpression of *HuERF1* reduced ROS damage in *Arabidopsis* by decreasing H_2_O_2_ and O_2_$\bar{\cdot }$ under salt stress.

To explore the possible molecular mechanisms of *HuERF1* under salt stress, the expression levels of several oxidation-related genes were analyzed by qRT-PCR under 300 mM NaCl treatment (Figure 6E–H). Although the expression of the ROS scavenging related genes (*APX2*, *CAT1*, *CSD1*, and *FSD1*) was increased in both WT and OE lines, the expression in the OE line was much higher than that in the WT under the salt treatment. These results indicated that overexpression of *HuERF1* in *Arabidopsis* could promote the expression of ROS scavenging-related genes and then possibly enhance *Arabidopsis* tolerance to salt stress.

## 3. Discussion

In this study, an AP2/ERF transcription factor, HuERF1, was characterized in pitaya. The Ap2/ERF family plays a key role in regulating plant growth, development, and the response to various abiotic stresses [25]. Within the ERF family, the ERF and DREB subfamilies have been distinguished by two conserved amino acid residues in the AP2/ERF domain depending on the identity of residues at specific positions [16]. That is, the 14th alanine and 19th aspartate positions are conserved in the ERF domain, whereas the valine and glutamate residues are conserved at the corresponding positions of the DREB domain. Similar to other ERF proteins, the HuERF1 protein has the 14th alanine and 19th aspartate positions in the 58-amino acid AP2/ERF domain. ERF proteins have been shown to act as repressors or activators depending on whether they suppress or activate the transcription of specific target genes. Tobacco NtERF2/4/98, *Arabidopsis* AtERF1/2/5, periwinkle ORCA2/3, and tomato Pti4 function as activators of transcription [26,27,28,29,30]. Transcriptional activation assay showed that HuERF1 exhibited transcriptional activation activity and that transcriptional activation domain was located at the C-terminus of the protein, however, the rest parts of the protein have hardly transcriptional activity (Figure 5A,B) and the recombinant HuERF1 protein was localized in the nucleus of *Arabidopsis* protoplasts (Figure 5C). The function of the N-terminal MCGGAII/L motif is unknown. However, it may not be necessary for nuclear localization [31]. This N terminal motif was found only in the ERF proteins. The phylogenetic tree analysis revealed that HuERF1 shares 53.82% identity with LeERF2 in tomato plants (Figure 1B). Therefore, the HuERF1 protein belongs to a member of the ERF subfamily.

Soil salinity is one of the key factors that inhibits the productivity and quality of crops. Overexpression of salt tolerance-related genes is an important strategy for increasing salt tolerance in crops. Expression analysis of *HuERF1* showed much higher expression level in the roots of pitaya than in its stem, petal, calyx, and squama (Figure S1), similar to that reported in *Fraxinus mandshurica* [32]. It was observed that *ERF84* transcripts in tomato were detected in breaker fruits and flowers but not in roots [33]. This indicates that the *ERF* genes have different expression patterns in plants. Moreover, the expression of *HuERF1* was rapidly induced by salt stress and peaked at 12 h of salt stress (Figure 3B). Similar findings were reported previously for sweet potato [17], pepper [34], and *Fraxinus mandshurica* [32]. In maize, the expression of *ZmERF1* was significantly increased by high salinity, heat, and osmotic stresses [22]. In cotton, *GhERF4L* and *GhERF54L* were significantly induced by salinity and played a key role in salt tolerance [35]. In tomato, *LeERF1-* and *LeERF2*-overexpressing transgenic plants had increased tolerance to salt stress [36]. Therefore, it has been proved that the *ERF* genes, including *HuERF1*, could play important roles in response to salt stress.

It is known that ethylene is an important inducer of defense-related genes in plants. In soybean, the expression level of *GmERF3* was induced by high salt, drought, and exogenous hormones including ABA, salicylic acid (SA), ethylene, and jasmonic acid (JA) treatments. The overexpression of *GmERF3* in tobacco plants enhanced tolerance to high salinity and drought [37]. *IbRAP2-12* was induced by NaCl, polyethylene glycol (PEG), ABA, ethylene, and methyle jasmonate (MeJA). *IbRAP2-12*-overexpressing *Arabidopsis* plants had increased tolerance to salt and drought stress [17]. In this study, expression of *HuERF1* was induced not only by high salinity but also by ET (Figure 3C). Since *HuERF1* increased salt stress tolerance and was induced by ethylene, it is hypothesized that *HuERF1* might be involved in salt stress tolerance by modulating the ethylene response. To provide evidence for this possibility, the ethylene-mediated salt stress tolerance was assessed when we treated pitaya seedlings with ET or 1-MCP as well as 600 mM NaCl (Figure 2C,D). Ethylene could further enhance the tolerance of piyata seedlings to salt stress (Figure 2D). These results indicated that *HuERF1* may play key roles in ethylene-mediated salt tolerance. But the survival rates of the piyata seedlings under NaCl as well as 1-MCP had no significant difference with those under NaCl (Figure 2G), which may be 1-MCP, does not easily enter the piyata seedlings or low dosage of 1-MCP was used.

Overproduction of ROS in plants is caused by salinity and drought and causes damage to macromolecular compounds, ultimately leading to oxidative stress [24]. ROS can be maintained at a low level by the ROS-scavenging pathway [38]. H_2_O_2_ is one of the key ROS. Increasing the expression level of genes involved in the ROS-scavenging pathway can decrease the accumulation of H_2_O_2_. In *Arabidopsis*, both *cat3* and *cpk8* mutants had higher accumulation of H_2_O_2_ and showed a drought-sensitive phenotype compared to the WT [38]. In this study, the accumulation of O_2_$\bar{\cdot }$ and H_2_O_2_ was lower in *HuERF1*-overexpressing *Arabidopsis* plants than in the WT under salt stress (Figure 6). In addition, several ROS-scavenging genes including *APX2*, *CAT1*, *CSD1*, and *FSD1* were upregulated in transgenic lines under salt stress (Figure 6). Therefore, the enhanced salt tolerance in transgenic *Arabidopsis* lines might be due to the enhanced ROS-scavenging potential [39].

## 4. Materials and Methods

### 4.1. Plants and Growth Conditions

Seeds of red pitaya were germinated and cultivated in a greenhouse (16 h/8 h, light/dark photoperiod, 25 °C). *Arabidopsis thaliana* (Col-0) was cultivated in a fixed greenhouse (16 h/8 h, light/dark photoperiod, 22 °C).

### 4.2. Sequence Analysis of HuERF1

Protein sequence alignment of HuERF1 and other ERF-like proteins was carried out using DNAMAN 7.0 software (Lynnon Biosoft Corp., San Ramon, CA., USA). The neighbor joining (NJ) method was used to construct the phylogenetic tree of pitaya HuERF1 by MEGA 7.0 software [40]. Bootstrap values were assessed (with 1000 replicates) to evaluate the relative support for each branch. The conserved domain of the HuERF1 protein was analyzed by the Smart program (http://smart.embl-heidelberg.de/). The nuclear localization signal of the HuERF1 protein was predicted by the cNLS mapper program (http://nls-mapper.iab.keio.ac.jp/cgi-bin/NLS_Mapper_form.cgi).

### 4.3. Vector Construction and Genetic Transformation

To generate the recombinant vector for the overexpression assay in transgenic *Arabidopsis*, the open reading frame (ORF) of the *HuERF1* gene was cloned into the pCAMBIA1302-v vector (modified from pCAMBIA1302) to generate *35Spro*::*HuERF1* recombinant plasmids. After sequencing confirmation, the constructed vector was transferred into *Agrobacterium tumefaciens EHA*105 and then transformed into *Arabidopsis* using the floral-dip method [41]. Overexpressing plants were screened on MS medium supplemented with 50 µg/L kanamycin according to the segregation ratio (sensitive: resistant = 1:3) and confirmed by PCR with the primer pair *HuERF1*-F/R. The expression levels of *HuERF1* were detected using qRT-PCR analysis as described above. The primers of *HuERF1*and *HuEF1-α* are listed in Table S1.

### 4.4. Transactivation Analysis and Subcellular Localization of HuERF1

The full-length ORF of *HuERF1* and five truncated *HuERF1* fragments were cloned into the pGBKT7 (Clontech, Mountain View, CA, USA). For HuERF1 transactivation assays, the constructed vectors, positive control pGBKT7-AtERF1, and empty vector pGBKT7, were transformed into *Saccharomyces cerevisiae* strain AH109. The yeast constructs were incubated in liquid SD-1 medium to OD_600_ 1.0, and then they were diluted using a gradient dilution (1:10, 1:100, and 1:1000). The transformed yeast strains were spotted on SD/-Trp and SD/-Trp/-His medium plates for 4 days at 30 °C.

The complete coding sequence of *HuERF1* was fused to the N-terminus of mGFP under the control of the *CaMV 35S* promoter. The fusion construction (*HuERF1-GFP*) and the empty vector were transformed into *Arabidopsis* protoplasts. GFP fluorescence in *Arabidopsis* protoplasts was observed under a laser confocal scanning microscope (LSM510, Zeiss, Jena, Germany) at an emission wavelength of 500 ± 50 nm and at an excitation wavelength of 488 nm.

### 4.5. Stress Treatment of Pitaya Plant

Pitaya seedlings were planted in pots containing nutrient soil and vermiculite (3:1) and grown in green house (16 h/8 h, light/dark photoperiod, 25 °C), and then subjected to salt stress. For the various treatments, two-week–old pitaya seedlings were dipped into different concentrations of NaCl solution (0–800 mM) for salt stress treatment for 15 days. Six-week-old pitaya seedlings were treated with 600 mM NaCl as well as 100 µM ET or 100 µM 1-MCP in an airtight container for 30 days. Survival rates and plant heights were measured.

### 4.6. Performance of Transgenic Arabidopsis under Salt Stress Treatments

For the assay of seed germination rates, seeds (> 50) from WT plants and transgenic *Arabidopsis* homozygous T_4_ plants were cultivated on MS medium containing 0 or 150 mM NaCl for 6 days. The germination rates were calculated after seeding. 

In vitro assay, five-day-old *Arabidopsis* seedlings were cultivated on MS medium containing 0 or 100 mM NaCl for 15 days. The root length and survival rates were measured after 15 days.

In the salt stress assay, three-week-old transgenic and WT seedlings were grown in pots containing a mixture of soil, vermiculite, and humus (1: 1: 1, *v*/*v*/*v*). The plants were watered with a 300 mM NaCl solution every three days for 3 weeks to simulate salt stress. Survival rates were measured.

### 4.7. RNA Isolation and qRT-PCR Analysis

Total RNA of aerial parts of pitaya and *Arabidopsis* plants were extracted using Eastep Super Total RNA Extraction Kit (Promega, Beijing, China). Then the residual genomic DNA was removed with RNase-free DNaseI (Promega, Beijing, China). The first-strand cDNA was synthesized from 1μg of DNA-free RNA using a GoScript^TM^ Reverse Transcription Mix (Promega, Beijing, China) in a 10 µL reaction volume according to the manufacturer’s instructions. The synthesized cDNAs were diluted five-fold for qRT-PCR assay. qRT-PCR was carried out with Eastep qPCR Master Mix Kit (Promega, Beijing, China) using Roche Light Cycler 480 Real-time PCR System (Roche, Basel, Switzerland). And dissociation kinetic curves were conducted according at the end of each qPCR run. All reactions were executed in triplicate for three biological replicates.

### 4.8. Histochemical and Physiological Analysis

In situ detection of superoxide (O_2_$\bar{\cdot }$) anion and hydrogen peroxide (H_2_O_2_) was determined with 1 mg/mL DAB or 1 mg/ml NBT solution for 10 h, and washing in 95% ethanol, respectively, according to the procedure of Kumar [42]. Superoxide dismutase (SOD) and catalase (CAT) activities were determined using SOD, and Catalase Assay Kits (Nanjing Jiancheng, Nanjing, China), according to the manufacturer’s instructions, respectively.

### 4.9. Statistical Analysis

IBM SPSS Statistics software (version 20.0, IBM Corp., Armonk, NY, USA) was used for statistical analysis. The value *p* < 0.05 or *p* < 0.01 was considered to be significantly different. All experiments were repeated at least three times.

## 5. Conclusions

Ethylene might enhance salt tolerance in pitaya. Pitaya HuERF1 is located in the nucleus and transcriptional activity. Overexpression of *HuEFF1* could enhance salt tolerance in transgenic *Arabidopsis*, and this overexpression in *Arabidopsis* may promote the expression of stress-related genes by causing a significant reduction in ROS accumulation. In summary, *HuERF1* is a positive ethylene regulator in plants for tolerance to salt stress, and it may be an ideal candidate to enhance salt tolerance in pitaya breeding.

## Supplementary Materials

Supplementary materials can be found at https://www.mdpi.com/1422-0067/21/13/4586/s1.

## Abbreviations

POD	Peroxidase
SOD	Superoxide dismutase
qRT-PCR	Quantitative real-time reverse transcription PCR
GFP	Green fluorescent protein
ORF	Open reading frame
ET	Ethephon
1-MCP	1-methylcyclopropene
CAT	Catalase
ROS	Reactive oxygen specie
O_2_$\bar{\cdot }$	Superoxide anion
APX1	Ascorbate peroxidase 1
CSD1	Copper/zinc superoxide dismutase
FSD1	Fe super dismutase 1
NBT	Nitrotetrazolium blue chloride
DAB	3,3’-Diaminobenzidine
