# Supplementary material for: An AP2/ERF Gene, HuERF1, from Pitaya (Hylocereus undatus) Positively Regulates Salt Tolerance
Source: Int J Mol Sci. 2020 Jun 28;21(13):4586. doi: 10.3390/ijms21134586 (PMC7369839; doi:10.3390/ijms21134586)
Supplement: Supplementary file 1 [file ijms-21-04586-s001.pdf]

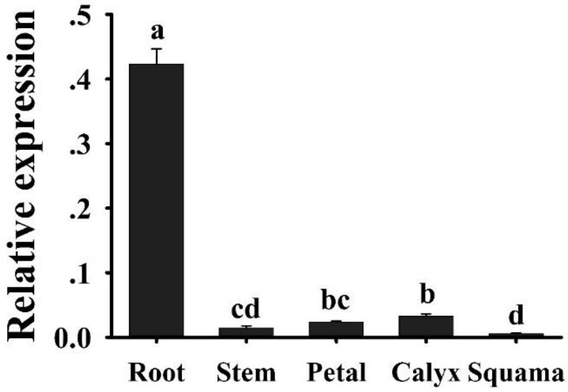

Figure S1. Differential expression of *HuERF1* in various tissues (root, stem, petal, calyx and squama). Bars with different letters indicate significant difference at $p < 0.05$. Mean values and SDs at least for three biological replicates are shown.

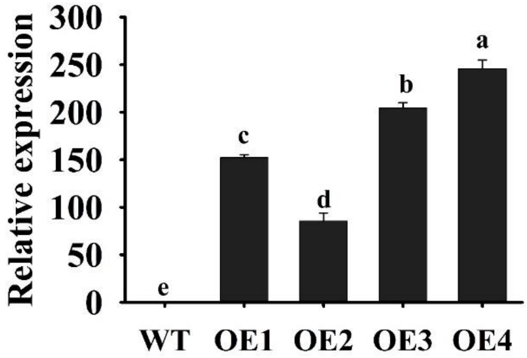

Figure S2. Differential expression of *HuERF1* in *HuERF1* overexpressing transgenic *Arabidopsis* lines and WT. Bars with different letters indicate significant difference at $p < 0.05$. Mean values and SDs at least for three biological replicates are shown.

Table S1. Primers used in this study.

Primer Name	Primer Sequence (5'–3')
Primers for constructing expression vector	
HuERF1-OE-F	TGACCATGGTAGATCTGATGTGTGGCGGTGCAATCTT
HuERF1-OE-R	CTTCTCCTTTACTAGTAGCAGCAGAAGAAGGAACATC
Primers for transactivation assay of *HuERF1*	
GAL4BD-HuERF1-F	GGAATTCATGTGTGGCGGTGCAATCTT
GAL4BD-HuERF1-R	AACTGCAGTTAAGCAGCAGAAGAAGGAAC
GAL4BD-HuERF1-N-F	GGAATTCATGTGTGGCGGTGCAATCTT
GAL4BD-HuERF1-N-R	AACTGCAGCTGCGTCTTCCTCTGCTTCT
GAL4BD-HuERF1-C-F	GGAATTCAGGAGACGACGTCGTTTAC
GAL4BD-HuERF1-C-R	AACTGCAGTTAAGCAGCAGAAGAAGGAAC
BD-HuERF1-DO-N-F	GGAATTCATGTGTGGCGGTGCAATCTT
BD-HuERF1-DO-N-R	AACTGCAGATTGGGGAAGTTCACCTTGG
BD-HuERF1-DO-C-F	GGAATTCTACAGAGGGATCCGGCAG
BD-HuERF1-DO-C-R	AACTGCAGTTAAGCAGCAGAAGAAGGAAC
BD-HuERF1-DO-F	GGAATTCTACAGAGGGATCCGGCAGCGA
BD-HuERF1-DO-R	AACTGCAGATTGGGGAAGTTCACCTTGGC
Primers for qRT-PCR	
HuERF1-RT-F	GCCTGTGACCCAGCAAGTGA
HuERF1-RT-R	CATCCAGATAGGGAAGTTCGTAGAA
HuEF1-α - F	CCCTGGTCAGATTGGAAACG
HuEF1-α - R	TCCCTCACGGCAAAACGA
CAT1-F	CGCCATGCCGAAAAATACCC
CAT1-R	CTTGCCTGTCTGAATCCCAGGAC
CSD1F	TGATGGAACTGCCACCTTCACA
CSD1R	ATGGCCTCCCTTTCCGAGGT
FSD1F	GCTCGGCTCTTTCCCATTGC
FSD1R	CAGCTTCCCAAGACACAAGATTGG
APX2-F	GGACGATGCCACAAGGAT
APX2-R	CGACCAAAGGACGGAAAA
AtActin2-F	GGTAACATTGTGCTCAGTGGTGG
AtActin2-R	AACGACCTTAATCTTCATGCTGC
